# Education and the improvement of clinical ethics services

**DOI:** 10.1186/1472-6920-13-41

**Published:** 2013-03-21

**Authors:** George J Agich

**Affiliations:** 1 Co-Director, International Conferences on Clinical Ethics & Consultation, 6805 Via Correto Dr, Austin, TX, 78749-2757, USA

**Keywords:** Clinical ethics, Ethics consultation, Ethics committees, Education, Quality improvement

## Abstract

The proliferation of clinical ethics in health care institutions around the world has raised the question about the qualifications of those who serve on ethics committees and ethics consultation services. This paper discusses some of weaknesses associated with the most common educational responses to this concern and proposes a complementary approach. Since the majority of those involved in clinical ethics are practicing health professionals, the question of qualification is especially challenging as the role of ethics committees and, increasingly, ethics consultation services are becoming increasingly important to the functioning of health care institutions. Since the challenging nature of health care finances often leads institutions to rely on voluntary participation of committed health professional with only token administrative or clerical support to provide the needed ethics services, significant challenges are created for attaining competence and functional effectiveness. The article suggests that a complementary approach should be adopted for sustaining and building capacity in clinical ethics. Ethics committees and consultation services should systematically adopt quality improvement techniques to effect designed changes in clinical ethics performance and to build ethical capacity within targeted clinical units and services. Demonstrating improvements in functioning can go a long way to build confidence and capacity for clinical ethics and can help in justifying the need for support. To do so, however, requires that ethics committees and consultation services first shift attention to those areas that demonstrate weak or questionable ethical performance, including the established practices of the ethics committee and consultation service, and second seek collaboration with the involved health care providers to pursue demonstrable change. Such an approach has a much better chance of improving the capacity for clinical ethics in health care institutions than relying on educational approaches alone.

## A commentary from George J Agich

The development and proliferation of healthcare ethics committees (HEC) and clinical ethics consultation services (ECS) is an international phenomenon that raises a common set of concerns about the preparation or qualifications of individuals to serve in such important functions. Although the numbers of health care professionals and non-health care professionals who have training in bioethics or clinical ethics are increasing, it is still a small percentage of those who serve on ethics committees and provide consultation services in patient care settings. Awareness of the problem of educational preparation for clinical ethics is hardly new. Publications like the *Improving Competencies in Clinical Ethics Consultation: An Education Guide* of the Clinical Ethics Task Force of the American Society for Bioethics and Humanities (ASBH) [1], for example, demonstrate that there is attention to the provision of education in support of these important functions. Nevertheless, it is fair to say that the need for education in clinical ethics has not been a high priority of bioethics programs or residency training programs [2-4]. Even so, many HECs devote significant portions of regular meetings to self-education and they undertake retreats or organize conferences on ethics topics. Commendable as these efforts are, they leave many areas unaddressed.

The hard reality is that much education in clinical ethics occurs without significant institutional support or resources, a fact that attests to the strong commitment of the individuals serving in these capacities. A good example of this commitment and level of interest is the work of the Student Clinical Ethics Committee at Kings College London, which addresses the important need of nurturing interest and providing early career training in clinical ethics [5]. Nonetheless, the primary weakness of current approaches is that clinical ethics education occurs at the margins of patient care and frequently focuses on ethical issues or topics that are of current or parochial interest rather than on subjects designed to address specific weaknesses or areas in which improvement in the provision of clinical ethics services is needed. As is known from many studies of organizational performance, reliance on formal educational programs, which are especially challenging in busy clinical settings, leads, at best, to evolutionary change.

It might reasonably be objected that the above claims are easy to make, but without foundation. In fact, there are no systematic studies of the educational preparation of HECs. To fairly judge whether the current state of clinical ethics education is satisfactory, one needs a matrix that identifies the fundamental or core areas of competence needed for serving on HECs or ECSs. Such a matrix will include not only cognitive areas, such as knowledge of ethical theory and concepts, applicable medical law and regulation, hospital policies, medical terminology, and a basic understanding of the organizational structure of the clinical settings, but also competence in interviewing, conflict or dispute resolution, and communication. Such a framework is implicit in the ASBH Clinical Ethics Task Force’s Education Guide [1], which also recognizes that more specialized knowledge and competences are required for dealing with the different challenges that arise in diverse clinical settings and institutions.

For example, research centers or academic medical centers that provide care for the sickest patients and deliver the most specialized or innovative interventions pose challenges that are quite different from those that are encountered in community or general hospitals. Hence, the knowledge base prerequisite for effective clinical ethics in such institutions is arguably different, even though there are obvious overlaps such as in policy development and ethics consultation as well as end of life care and communication conflicts. Nevertheless, institutional size, medical complexity and diversity of patient populations served, as well as the level of support of key administrators, medical, nursing, and allied health staffs are key factors that limit the scope of clinical ethics activities. Hence, these factors shape the educational prerequisites for the effective functioning of HECs and ECSs.

In particular, ECSs are sensitive to the types of ethical questions and issues that arise and recur in patient care on specialized units or wards. While problems surrounding end-of-life care are commonplace and so constitute a core topic in which ethics committee members and ethics consultants require competence, specialized care settings such as critical care units pose unique problems that are frequently intertwined with the communication style of physicians, nurses, and key allied health professionals on these units [6]. Even when the presenting issues involve the limitation of life-sustaining interventions, they are often laced with complex communication problems, which require ethics consultants serving these units to have more developed skills and knowledge [7].

A complementary approach to addressing the areas of weakness would be to apply quality improvement techniques in clinical ethics. This may present opportunities for much faster and more lasting learning. Since clinical ethics is a practical ethics applied to problems arising in patient care, theoretical ethics, though important, needs to be augmented by the honing practical skills in handling the unique ethical problems arising in specific patient care settings. To implement quality improvement in clinical ethics, hospital ethics committees and ethics consultation services alike need to focus on identifying areas of weakness rather than touting strengths and successes. Many surveys of clinical ethics services, for example, are designed to address satisfaction rather than to identify problems and weaknesses that can be used to formulate action plans using quality improvement methodology. For example, ethics committees often complain that ethical problems in patient care occur, but that the committee or consultation service is not involved.

This commonly identified challenge is often addressed by formal education about ethics consultation services with limited success. Since the obstacles to the acceptance of ethics consultation are complex and are institution and unit specific, formal education should be regarded as only one component of a larger effort at institutional change. A good example of this is the common problem of lack of requests for clinical ethics consultation. Standard quality improvement approaches can be used to make structural changes designed to improve access to ethics services. Units with special problems such as critical care units can be targeted for ethics liaison services, which have the advantage of familiarizing staff with clinical ethics consultation and for recognizing them as collaborating consultants rather than as ethics “police,” who are the very last resort for mediating conflict [8]. Such an approach has a much better likelihood of success than simple education of health care professionals about the availability of services. The problem is often not ignorance of the existence of the ethics support services, but rather the perception of its unavailability at the time of need or the slow response to requests and poor follow up of cases. Each of these weaknesses can be identified and are amenable to organizational performance improvement approaches. HECs and ECSs need to accept that the very problematic behaviors of healthcare professionals and patterns of health care delivery are can be a vital resource for clinical ethics to bring effective ethical improvements into patient care.

## Competing interests

The authors declare that they have no competing interests.

## Pre-publication history

The pre-publication history for this paper can be accessed here:

http://www.biomedcentral.com/1472-6920/13/41/prepub
